# Modeling the Articular Surface of the Hamate with the Fourth and Fifth Metacarpal Bases with Three-dimensional Laser Scanning

**DOI:** 10.7759/cureus.6447

**Published:** 2019-12-22

**Authors:** Matthew C McRae, Stephanie Dreckmann, Sandeep S Sandhu, Paul Binhammer

**Affiliations:** 1 Plastic Surgery, St. Joseph's Hospital, McMaster University, Hamilton, CAN; 2 Plastic Surgery, University of Toronto, Toronto, CAN; 3 Obstetrics and Gynecology, University of Toronto, Toronto, CAN; 4 Plastic Surgery, Sunnybrook Health Sciences Center/University of Toronto, Toronto, CAN

**Keywords:** hamate, carpo-metacarpal, 3d laser scan, articular surface, metacarpal, modelling

## Abstract

Introduction

Our purpose is to highlight the articulating surfaces between the hamate and fourth and fifth metacarpal (MC) bases of the hand using three- dimensional (3D) laser scanning. This joint surface is used for osteochondral grafting of small joints such as the proximal interphalangeal joint using the hamate articular surface. It is an important joint for hand function and can develop osteoarthritis.

Methods

NextEngine (NextEngine, Santa Monica, CA) 3D laser scanner (accurate to ±100 µm) was used to capture the articular surfaces of the hamate with the fourth and fifth MC bases of 10 embalmed cadaver right hands. Articular surfaces were defined and modeled using Amira (Visage Imaging, Andover, MA) and MatLab7 (MathWorks, Natick, MA). Articular surfaces were evaluated in terms of size, shape, the radius of curvature (ROC) by three points and sphere-fit (SF) and inter-facet angles.

Results

In the fourth carpometacarpal (CMC) joint, the hamate articular surface with the 4th MC was single, concave, and well approximated by SF ROC (mean: 11.18 mm). The fourth MC base was convex; SF ROC mean was 9.94 mm. Six of the 10 articulations flattened from volar to dorsal.

In the fifth CMC joint, we noted a bicondylar construct. The two hamate surfaces were concave while MC bases were convex. The joint surface was best approximated with two overlapping spheres. Ulnar sphere averaged 30.21% of the surface of the hamate and 29% of the MC base. Ulnar hamate SF ROC mean was 11.63 mm, and ulnar fifth MC SF ROC mean was 8.07 mm. Radial SF hamate mean was 7.92 mm, and the radial fifth MC SF mean was 7.47 mm. The mean of the angle of divergence between the condylar spheres represented on the hamate surface was 21.4°, while that of the fifth MC base angle of divergence was 10.99°.

The mean of the angle formed between the fourth and fifth CMC joints at the hamate was 31.69°. A single articular facet between the fourth and fifth MC bases was concave on fourth and convex on the fifth MC base.

Conclusions and clinical relevance

Laser scanning of cadaver fourth and fifth CMC joints clarified the normal anatomy of the osteochondral joint surface. The topography of the joints was well-approximated by SF with curved surfaces in both the anteroposterior and radial-ulnar planes with the fifth CMC having two unique surfaces for articulation. We noted the distinct radial and ulnar articulating surfaces of the fifth CMC joint, which would permit flexion and limited supination.

## Introduction

There is a paucity of literature describing the morphology of the articular surface of the hamate with the fourth and fifth metacarpal (MC). Reasons for this include the small surface area and limited accuracy and precision of imaging; additionally, in clinical terms, injuries to the hamate seem to only minimally affect long-term hand function [1-3]. A perceived lack of morbidity and purported contour similarity have led to the use of the dorsum of the hamate as an expendable osteocartilaginous autograft [3,4]. Hastings first recommended the hemi-hamate autograft for the repair of the palmar base of the middle phalanx (podium presentation: Hastings H, Capo J, Steinberg B, Stern P. Hemicondylar Hamate Replacement Arthroplasty for Proximal Interphalangeal Joint Fracture/Dislocations. 54th Annual Meeting of the American Society for Surgery of the Hand; Boston, MA. September 2-4, 1999). The procedure, which replaces the damaged palmar lip of the middle phalanx with a size-matched segment of the dorsal distal surface of the hamate between the fourth and fifth MCs, has demonstrated good clinical outcomes [4-9]. There is concern about the occurrence of osteoarthritis, attributable to an anatomical mismatch between the MC and the hamate [8-11]. A cadaveric investigation employing 3D imaging observed a lack of uniform morphologic similarity between the middle phalanx and the hamate [11]. This work, and others, suggests that the contour of the hamate may not achieve a stable, congruent repair of the proximal interphalangeal joint, and urges to be cautious of the use of the hemi-hamate osteocartilaginous autograft [4,9].

Furthermore, few investigations have assessed morbidity of the hamate and consequences to the articular surfaces of the fourth and fifth MC following graft harvesting. The morphology of the articular surface of the hamate with the fourth and fifth MC bases permits the motion and stability for specific hand functions. In particular, two unique human hand grips of precision (throwing) and power (clubbing) are only possible due to the unique morphology of this joint that combines stability, flexion, and supination [12].

The purpose of this descriptive study is to evaluate the articular surface of the hamate and the congruous surfaces of the fourth and fifth MC bases using novel technology and mathematical modeling to better describe morphology in vivo. These hypotheses-generating observations will hopefully improve our understanding of these joint surfaces as well as our overall understanding of the interface between hand and wrist anatomy. This study has been presented at a conference as a poster presentation, and an article version of it was later published in the Canadian Journal of Plastic Surgery [13]. 

## Materials and methods

Specimens

Ten embalmed human cadaver right hands with a mean age of 81 years (range: 65-91 years) were obtained for dissection. Specimens were stored at -25°C in vacuum-sealed bags and thawed 24 hours prior to dissection. The hamate fourth and fifth MCs were carefully dissected from soft tissue to preserve articular surface anatomy and disarticulated from the hand. Joint surfaces were inspected to ensure that they were free of underlying joint damage. Samples were stored in saline-soaked gauze to prevent moisture loss prior to 3D scanning. Scan time for each sample was approximately 35 minutes. Samples were scanned immediately after dissection.

A NextEngine 3D laser scanner (NextEngine, Santa Monica, CA) imaged the articulating surface of the hamate with the fourth and fifth MC bases according to methods described by Podolsky [14]. This system allows for highly accurate image acquisition (± 100 µm) with twin array of four class 1M, 10-mW solid-state lasers at 650 nm wavelength with twin 30-megapixel image sensors and optical seven-color capture. Specimens were prepared with four 2.5 mm K-wires mounted with fiducial reference markers for computational registration. Talcum powder spray was applied to assist in image capture. Specimens were mounted on a high-precision 2-axis turntable for multiple scans and processed in OpenGL 3D viewer, ScanStudio HD Pro software (NextEngine, Santa Monica, CA).

Materials for analysis

The articular surfaces were defined using Amira version 5.3.3 (Visage Imaging, Andover, MA). Measurements of each joint surface included the surface area, the length (which was calculated in the mid-sagittal plane), and the width (which was calculated in the mid-coronal plane).

The best-fit radius of curvature (ROC) was calculated for each concave or convex articular surface. The ROC calculates the sphere size that would represent the surface with the least variation from the actual surface represented. The error from the actual model surface was calculated as a mean error as a percentage of the radius calculated.

The relationships of the articular surfaces of the hamate and MC were calculated as the angle of divergence. All angles were calculated at the mid-coronal plane. MatLab 7.10 (The MathWorks, Natick, MA) was used for modeling the joint surface by best SF ROC and mean error of surface points to the modeled sphere. 

## Results

Articulation of the hamate with the fourth metacarpal base

A summary of measurements of the fourth carpometacarpal (CMC) joint is included in Table 1. Laser imaging and analysis showed an articular surface area of the hamate, which averaged 76 mm^2^ with (range: 63.02-89.32 mm^2^. This was compared with the base of the fourth MC surface area which averaged 79.42 mm^2 ^(range: 3.39-115.23 mm^2^). Mathematical modeling with the best SF ROC for the hamate articulation averaged 11.18 mm (range: 4.12-21.07 mm). The best SF ROC for the fourth MC base was 9.94 mm (range: 4.58-18.38 mm (Table 1). The mean error of each surface point to the best SF ROC was 0.34 mm (range: 0.19-0.86 mm). Significant flattening of the dorsal articulation on visual inspection in six of 10 specimens was correlated with a mean error of >0.25 mm.

**Table 1:** A summary of the measurements of the fourth carpometacarpal joint MC: metacarpal; CMC: carpometacarpal; ROC: radius of curvature; SF: sphere-fit

	Fourth MC base articular surface	Hamate fourth CMC articular surface
N = 10	Mean (range)	Mean (range)
Surface area (mm^2^)	79.42 (53.37–115.23)	76.03 (63.02–89.32)
Mid-sagittal length (mm)	11.79 (10.15–13.39)	11.36 (8.93–14.12)
Mid-coronal width (mm)	6.94 (5.71–9.26)	7.05 (6.06–8.55)
Best SF ROC (mm)	9.94 (4.59–19.82)	11.18 (4.12–21.07)
Error of each surface point to best SF ROC (mm)	0.33 (0.14–0.67)	0.34 (0.19–0.86)

**Figure 1:**
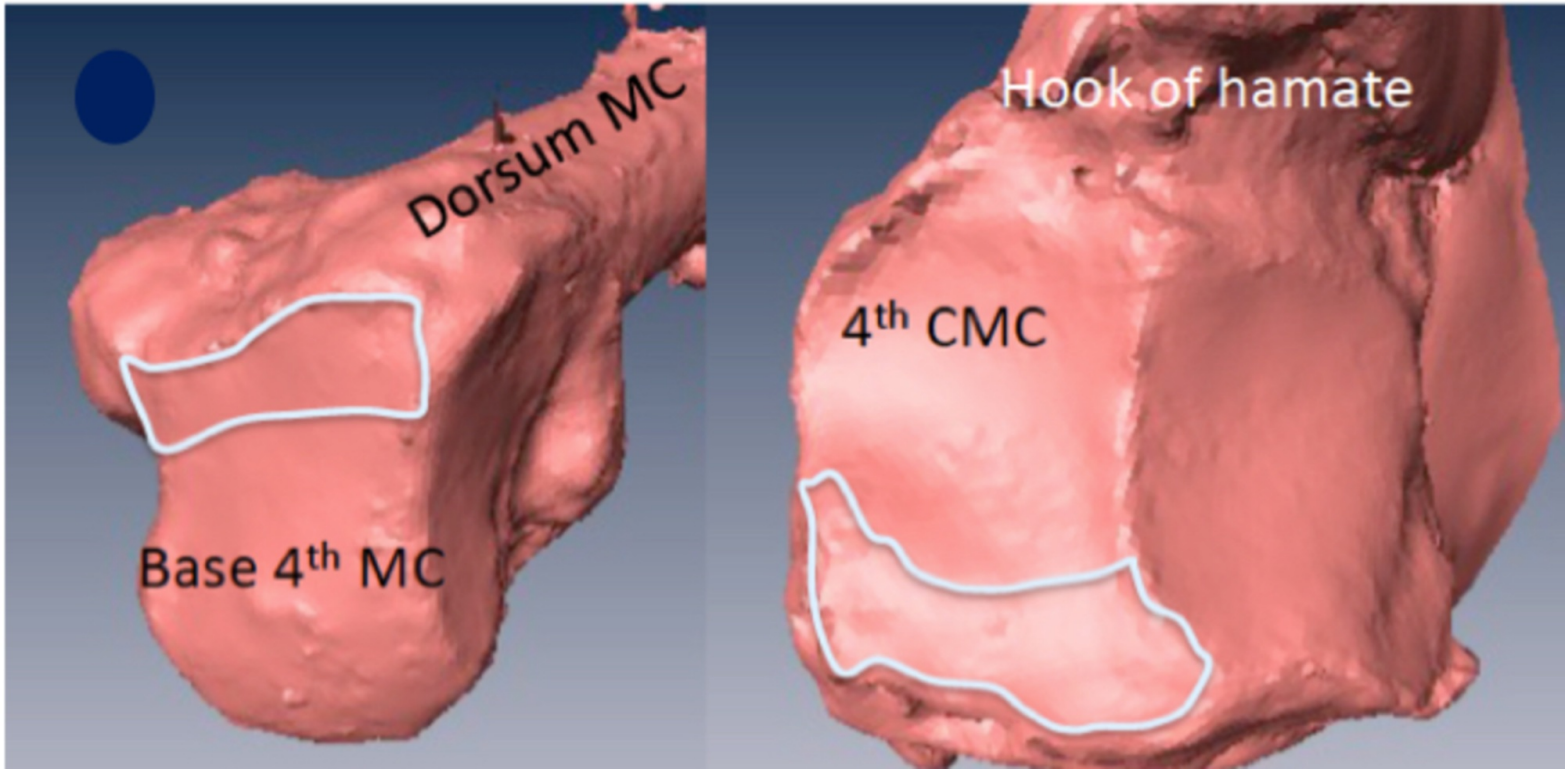
Six of 10 samples had dorsal flattening of the joint. The flat dorsal joint surface is outlined MC: metacarpal; CMC: carpometacarpal

Visual inspection revealed a concave joint surface on the hamate congruous with the convex fourth MC base. The dorsal articulation of the joint flattened in six of 10 specimens (Figure 1).

Articulation of the hamate with the fifth metacarpal base

**Figure 2:**
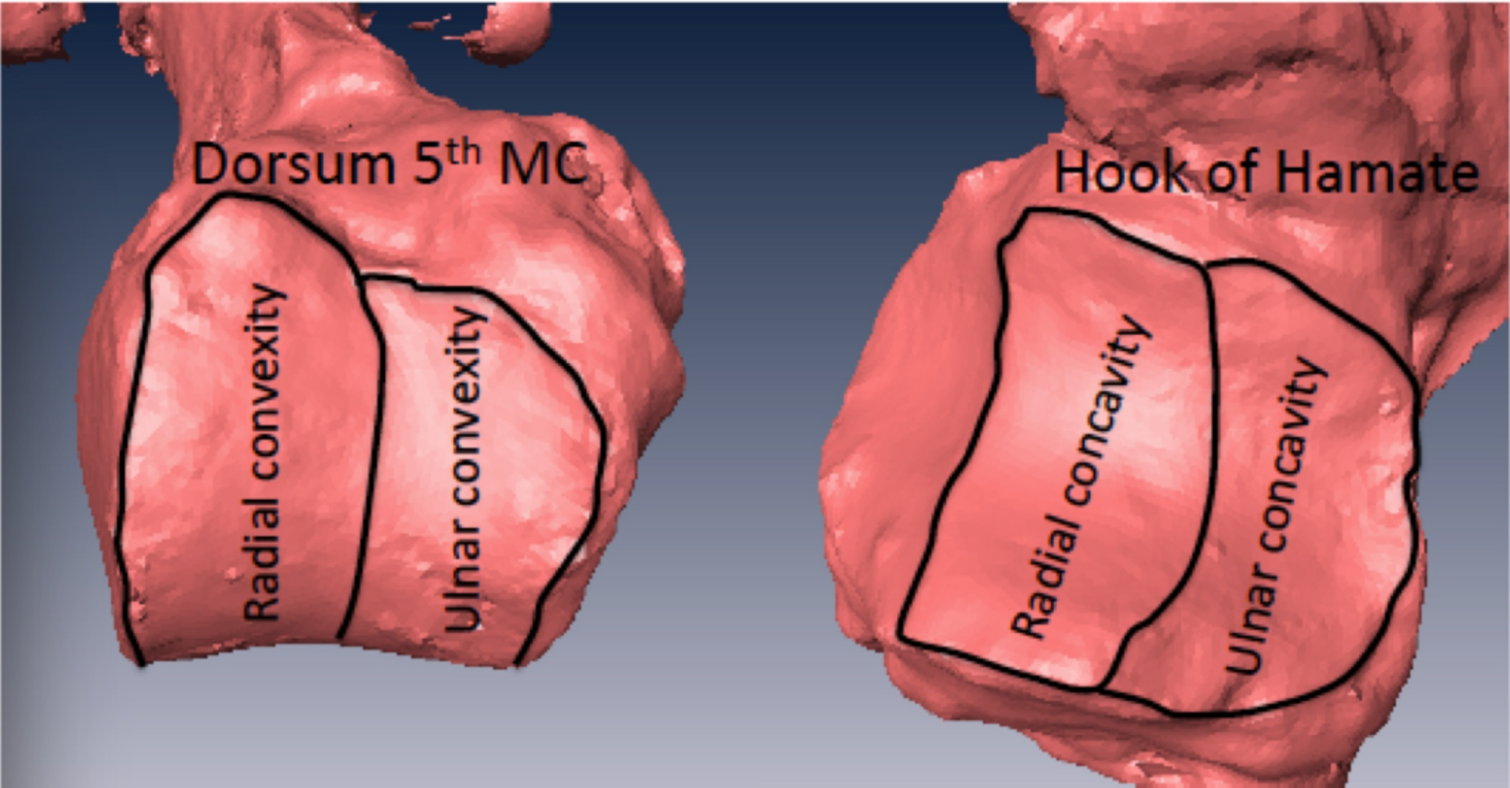
Representative samples of the base of the fifth metacarpal articular surface with its radial and ulnar convexities outlined. The radial and ulnar concavities of the congruent hamate surface is also outlined MC: metacarpal

Laser-assisted visual inspection with software analysis revealed a biconcave joint surface on the hamate congruous with the biconvex fifth MC base (Figure 2).

Laser imaging and analysis measured the average articular surface of the hamate to be 90.35 mm^2^ (range: 57.62-122.94 mm^2^). Detailed measurements are listed in Table 2. The ulnar concave surface of the hamate was on average 30.21% of the articular surface area of the fifth CMC joint (range: 19-51.14%). The fifth MC base surface area average was 111.45 mm^2^ (range: 87.01-155.32 mm^2^). The ulnar convex surface was on average 29% of the total articular surface area (range: 18.9-43.59%).

**Table 2:** A summary of the measurements of the fifth carpometacarpal joint MC: metacarpal; CMC: carpometacarpal

	Fifth MC base articular surface	Hamate fifth CMC articular surface
N = 10	Mean (range)	Mean (range)
Surface area (mm^2^)	111.41 (87.01–155.32)	90.35 (57.62–122.94)
Mid-sagittal length (mm)	13.73 (10.53–18.38)	10.59 (9.37–12.38)
Mid-coronal width (mm)	10.04 (8.16–12.35)	9.85 (8.05–12.67)

**Figure 3:**
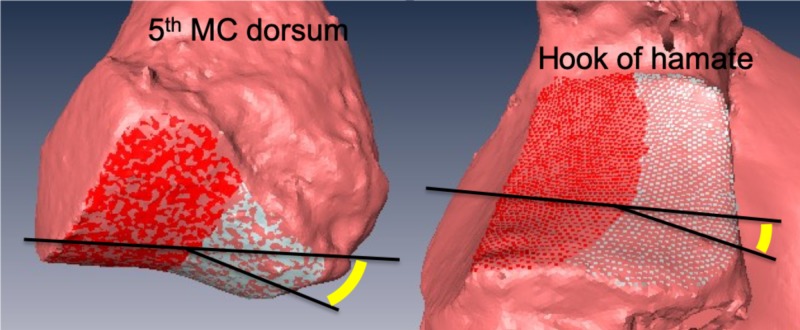
The angle of divergence between the ulnar and radial aspect of the fifth carpometacarpal joint MC: metacarpal The angle of divergence is symbolized in yellow

The angle of divergence between the ulnar and radial aspect of the fifth CMC joint was measured at 11° (range: 2-18.7°) at the MC base and 21.4° (range: 14.3-35.9°) at the hamate (Figure 3).

**Figure 4:**
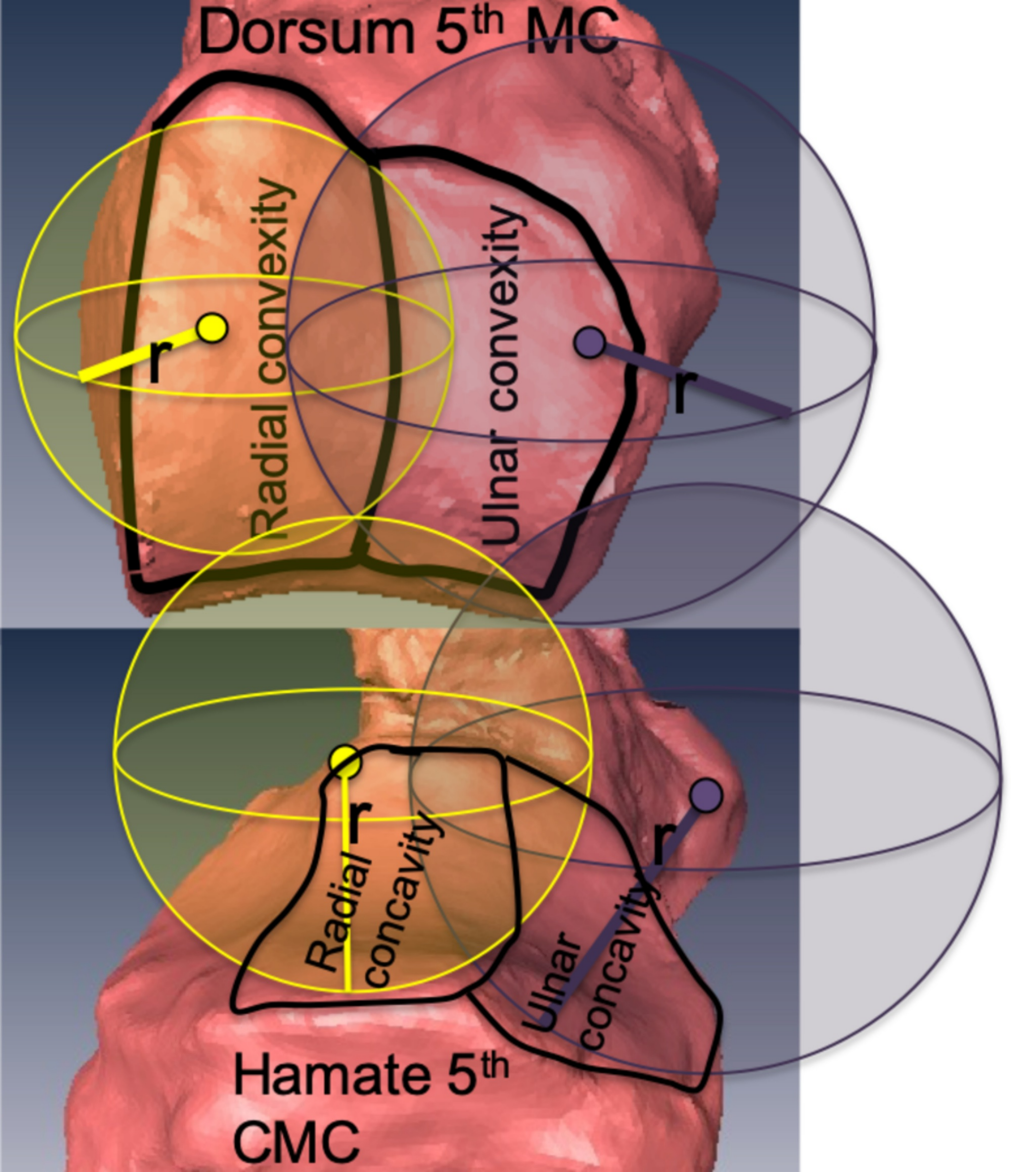
Best sphere-fit to characterize fifth carpometacarpal joint surfaces r: radius of curvature of the best sphere-fit model of the joint surface; the spheres model the two distinct joint surfaces on the base of the fifth metacarpal and the two distinct surfaces of the hamate that form the fifth carpometacarpal joint

Mathematical modeling with best SF ROC for the radial surface of the hamate averaged 7.92 mm (range: 5.99-10.82 mm; average point error: 0.22 mm) with the ulnar concavity averaging 11.63 mm (range: 7.75-18.27 mm; average point error: 0.13 mm). The radial convexity of the fifth MC base averaged 7.47 mm (range: 6.03-8.85 mm; average point error: 0.17 mm). The ulnar convexity of the fifth MC base average sphere fit was 11.63 mm (range: 7.74-14.28 mm; average point error: 0.13 mm) (Figure 4). The ROC was larger in all instances for the ulnar articulation of the fifth CMC joint (Table 3).

**Table 3:** Best sphere-fit for fifth carpometacarpal joint MC: metacarpal; CMC: carpometacarpal; ROC: radius of curvature; SF: sphere-fit

N=10	Fifth MC base articular surface		Hamate articulation with fifth CMC	
	Radial convexity	Ulnar convexity	Radial concavity	Ulnar concavity
	Mean (range)		Mean (range)	
Best SF ROC (mm)	7.47 (6.03–8.85)	8.07 (5.71–12.01)	7.92 (5.99–10.82)	11.63 (7.75–18.74)
Error of each surface point to best SF ROC (mm)	0.17 (0.11–0.23)	0.095 (0.048–0.162)	0.22 (0.14–0.34)	0.13 (0.0470.21)

Articulation between fourth and fifth carpometacarpal joints

**Figure 5:**
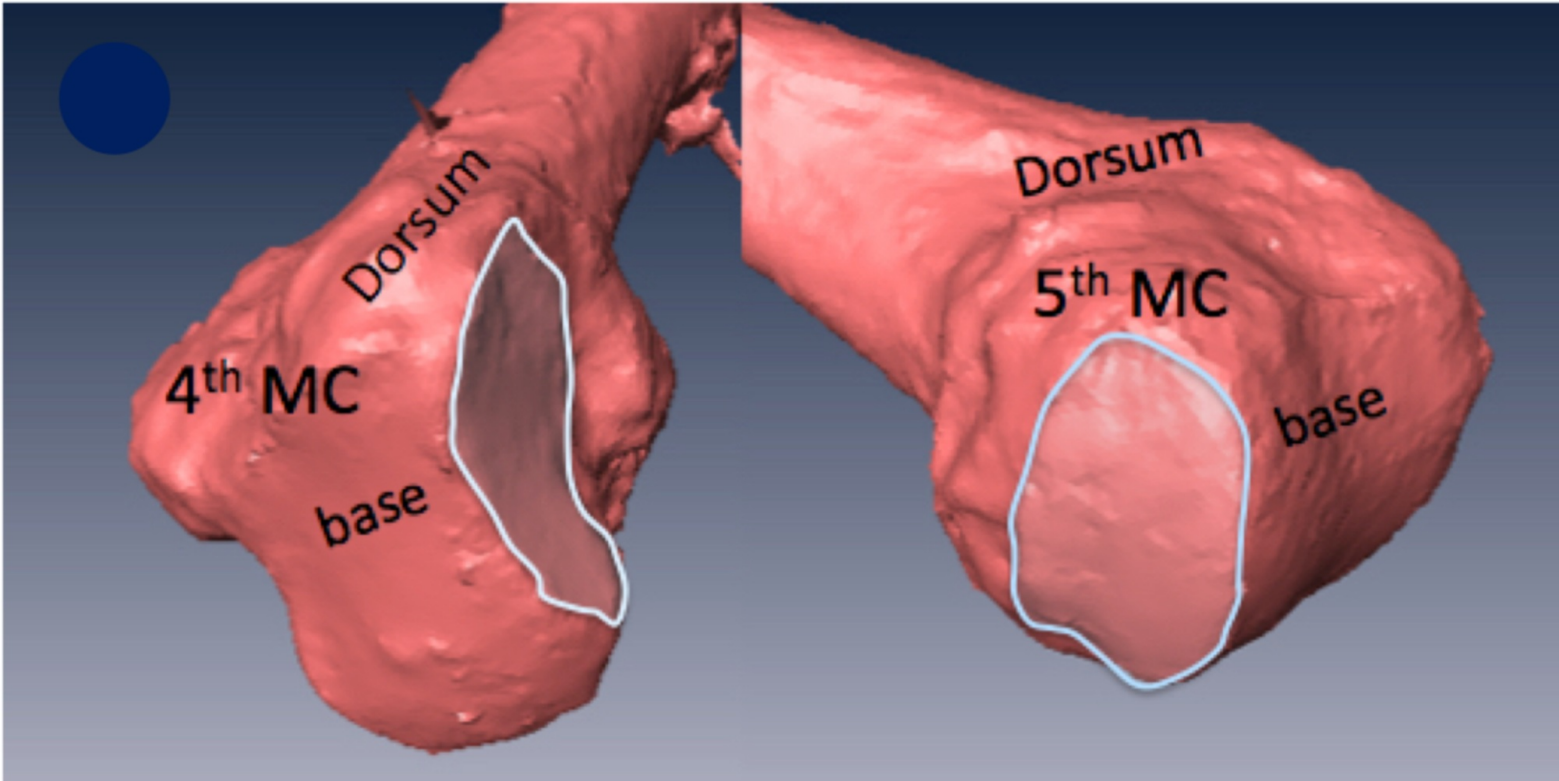
Articulation between concave fourth and convex fifth metacarpal bases Articulation between the fourth and fifth MC base outlined in blue MC: metacarpal

**Figure 6:**
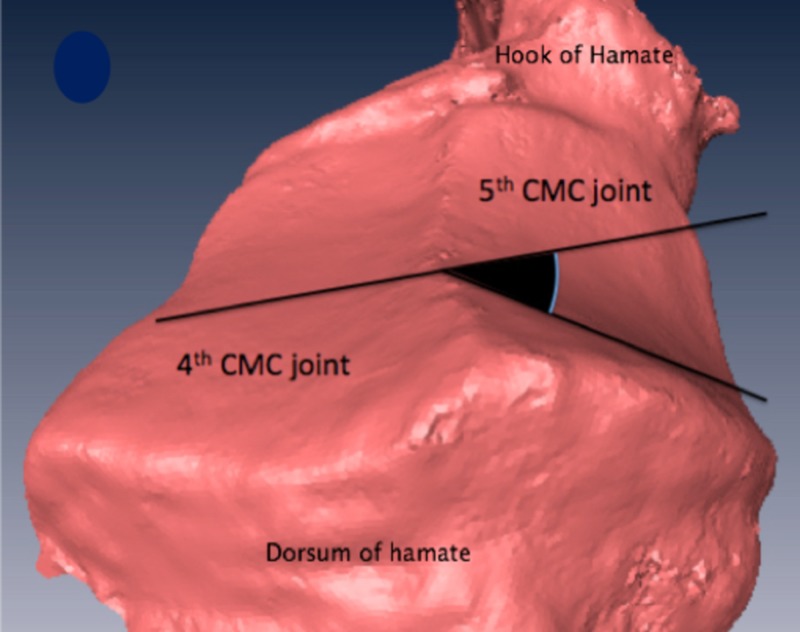
Mid-coronal angle on the articular surface of hamate between fourth and fifth carpometacarpal joints CMC: carpometacarpal; the angle formed between the joint surface of the fourth and fifth carpometacarpal joint on the hamate (given in black) was measured at the mid-coronal line of the joint surface

Between the bases of the fourth and fifth MCs, there was a consistent articular surface that was concave at the fourth MC and convex on the fifth MC base (Figure 5). The morphology was more approximated to that of a cylinder (vertically flat). A 3-point ROC was obtained thereafter at the mid-axial surface of the joint. The concave fourth MC surface averaged 10.41 mm (range: 6.97-19.2 mm). The convex fifth MC surface 3-point ROC averaged 9.66 mm (range of 7.9-18.37 mm). The hamate surface shows a prominent ridge delineating the border of the 4th and 5th MC base positions. The angle between the joint surfaces at the mid-coronal plane of articular surface of the hamate averaged 33.94° (range: 29.7-41°) as displayed in Figure 6.

## Discussion

This descriptive anatomic study aimed to characterize the articular surface of the hamate with the fourth and fifth MC bases using laser scanning and a mathematical model of sphere-fit to enhance our understanding of this joint in vivo. We did not include the ligamentous anatomy as this has been described in detail elsewhere[15]. Laser scanning, with 100 µm resolution, is superior for examining small joint surfaces to other imaging modalities such as the MRI. While many MRI scans have limits of resolution of 1 mm, which limits utility in analyzing small joint surfaces, significant advancements in MRI technology continue to evolve with specific sequence coil and 3 Tesla magnets are able to approach 160 µm resolution [16]. MRI is a promising modality for future investigation of small joint surfaces in vivo*.*

Our current knowledge of these articular surfaces is limited to a number of cadaver studies that visually inspected the joint surfaces. Viegas et al. explained that the fourth CMC joint is the most varied joint in terms of the osseous morphology of the CMC joints [17]. They specifically identified five types of articulations that were defined by the presence or absence of an articular surface between the 4th MC base with the capitate. Our sole focus in this study was on the interface between the fourth and fifth MC bases and the hamate that were the primary axial load-bearing surfaces.

Our investigation provides evidence that the stability of the fourth CMC joint in extension and axial load may be enhanced by the joint architecture of a flat dorsal surface, as seen in six of the 10 specimens. The remaining four had “ball in socket” configurations.

**Figure 7:**
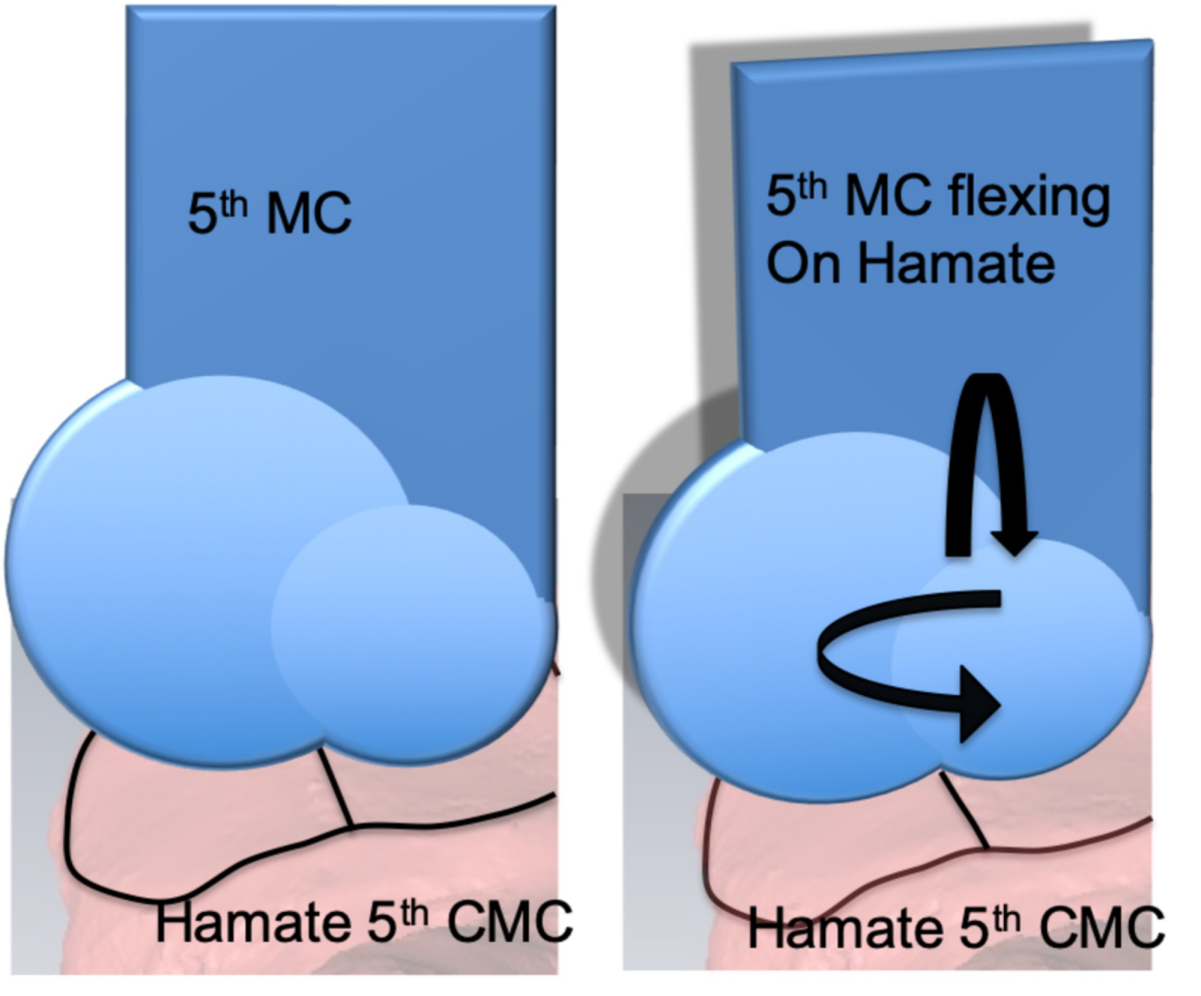
Fifth metacarpal flexing on biconcave hamate surface MC: metacarpal; CMC: carpometacarpal The base of the fifth metacarpal is modeled with best sphere-fit on the hamate surface. With flexion, a hinge joint could result in both flexion (vertical arrow) and an element of supination (horizontal arrow) due to differential sphere surface areas

Biomechanical studies showed that flexion at the fifth MC base may be as much as 35 degrees, but is most commonly quoted as being 10-15 degrees flexion with supination rotation after maximal flexion of 10-15 degrees [18,19]. Our observation of a biconcave surface to the hamate with the biconvex fifth MC base and a flatter ulnar surface could suggest improved stability on the ulnar aspect of the hand-wrist interface. This interface could be maintained while preserving the motion necessary for precision and power grip. The angle of divergence between the fourth and fifth MC joints is known to contribute to the digital cascade. In 1993, Bade et al. described the fifth CMC as saddle-shaped based on analysis of 50 cadavers, but presumed that the motion at the joint was mainly radial and ulnar [19]. In contrast, we feel that the angle of divergence between the two distinct articular surfaces of the fifth CMC joint and the differing ROCs promote supination with the flexion at the hamate. The fifth MC joint also contributes to the flexion cascade of the small finger to the scaphoid tubercle in full flexion (Figure 7). This would be a biomechanically more stable construct resembling a bicondylar joint at the CMC while permitting limited supination. Further in vivo evaluation would be necessary to prove either hypothesis.

The motion at the fourth and fifth CMC joints participate in both the precision and power grips of the hand. There is a sentiment among some hand surgeons that this joint universally does well post-injury, assuming anatomic alignment and stability. This appears to be supported by the use of the hemi-hamate autografts with limited morbidity, despite the removal of the dorsal half of the hamate articular surface [8,10,20]. Capo et al. recently assessed donor site stability following hemi-hamate graft harvesting. Despite five of the eight cadavers demonstrating statistically significant changes in subluxation following biomechanical testing, the authors reported that the donor site morbidity was clinically minimal [4]. In fact, donor site morbidity after hemi-hamate arthroplasty appears low, however, tenderness and nerve tethering in the scar have been reported, and long-term investigations are scarce [10,21-23]. Further, Sollaccio et al. observed substantial variation in articular surface morphology of the dorsal distal hamate between and within individuals, and no uniform similarity in shape between the articular surface of the dorsal distal hamate and the volar middle phalanx base [11]. As such, the variation in hamate morphology, the occurrence of osteoarthritis, and potential donor-site morbidity have led to investigations for alternative donor sites for volar middle phalanx reconstruction [10,14].

This study assists in the understanding of the bone and cartilaginous interface of the fourth and fifth CMC joints. However, it is not without limitations. The joint surfaces examined were from cadavers of advanced age. While we did not note evidence of osteoarthritis or cartilaginous loss at any of the joint surfaces, substantial alterations of anatomy could be a consequence of age-related changes. The substantial shape variation in the hamate articular surfaces likely requires a larger sample size in a more diverse patient population to improve the generalizability of the findings. Nonetheless, it serves to broaden the understanding of the fourth and fifth CMC joint, which serves not only as a donor site but is also associated with fracture dislocations.

## Conclusions

Laser scanning of the cadaver fourth and fifth CMC joints clarifies the normal anatomy of the osteochondral joint surface. The topography of the joints is well-approximated by sphere-fit with curved surfaces in both the anteroposterior and radial-ulnar planes. Six of the ten fourth CMC articular surfaces have a flat dorsal component that may stabilize the joint in extension and under axial load. There is a cylindrical articular surface between the fourth and fifth MC bases that may permit the supination of the fifth MC. The fifth CMC is broad and contains two distinct spherical surfaces analogous to a condylar joint that may provide stability in the radial-ulnar plane while also allowing flexion and extension. We believe our findings will hopefully improve the understanding of these joint surfaces as well as our overall understanding of the interface between hand and wrist anatomy.
